# First-attack pediatric hypertensive crisis presenting to the pediatric emergency department

**DOI:** 10.1186/1471-2431-12-200

**Published:** 2012-12-31

**Authors:** Wen-Chieh Yang, Lu-Lu Zhao, Chun-Yu Chen, Yung-Kang Wu, Yu-Jun Chang, Han-Ping Wu

**Affiliations:** 1 Department of Pediatrics, Changhua Christian Hospital, Changhua, Taiwan; 2 Department of Pediatrics, Buddhist Tzu-Chi General Hospital, Taipei Branch, Taichung, Taiwan; 3 Department of Surgery, Buddhist Tzu-Chi General Hospital, Taichung Branch, Taichung, Taiwan; 4 Laboratory of Epidemiology and Biostastics, Changhua Christian Hospital, Changhua, Taiwan; 5 Department of Pediatrics, Buddhist Tzu-Chi General Hospital, Taichung Branch, No.66, Sec. 1, Fongsing Rd., Tanzih Township, Taichung 42743, Taiwan; 6 Department of Medicine, Tzu Chi University, Hualien, Taiwan

**Keywords:** Hypertensive crisis, Children, Hypertensive urgency, Hypertensive emergency

## Abstract

**Background:**

Hypertensive crisis in children is a relatively rare condition presenting with elevated blood pressure (BP) and related symptoms, and it is potentially life-threatening. The aim of this study was to survey children with first attacks of hypertensive crisis arriving at the emergency department (ED), and to determine the related parameters that predicted the severity of hypertensive crisis in children by age group.

**Methods:**

This was a retrospective study conducted from 2000 to 2007 in pediatric patients aged 18 years and younger with a diagnosis of hypertensive crisis at the ED. All patients were divided into four age groups (infants, preschool age, elementary school age, and adolescents), and two severity groups (hypertensive urgency and hypertensive emergency). BP levels, etiology, severity, and clinical manifestations were analyzed by age group and compared between the hypertensive emergency and hypertensive urgency groups.

**Results:**

The mean systolic/diastolic BP in the hypertensive crisis patients was 161/102 mmHg. The major causes of hypertensive crisis were essential hypertension, renal disorders and endocrine/metabolic disorders. Half of all patients had a single underlying cause, and 8 had a combination of underlying causes. Headache was the most common symptom (54.5%), followed by dizziness (45.5%), nausea/vomiting (36.4%) and chest pain (29.1%). A family history of hypertension was a significant predictive factor for the older patients with hypertensive crisis. Clinical manifestations and severity showed a positive correlation with age. In contrast to diastolic BP, systolic BP showed a significant trend in the older children.

**Conclusions:**

Primary clinicians should pay attention to the pediatric patients who present with elevated blood pressure and related clinical hypertensive symptoms, especially headache, nausea/vomiting, and altered consciousness which may indicate that appropriate and immediate antihypertensive medications are necessary to prevent further damage.

## Background

It has been demonstrated that high blood pressure (BP) contributes to the early development of cardiovascular structural and functional changes in children [1,2]. With increasingly high BP, autoregulation eventually fails, leading to damage of the vascular wall and further organ hypoperfusion. Hypertensive crisis is a critical condition characterized by a rapid, inappropriate and symptomatic elevated BP, and is categorized as hypertensive urgency (without damage of target-organs) and hypertensive emergency, which is associated with rapid deterioration of target-organs (heart, brain, kidneys and arteries), and is a potentially life-threatening condition. Hypertensive encephalopathy, an example of hypertensive emergency, is associated with hypertension (HTN) and includes a combination of various neurological manifestations such as altered mental status, headache, nausea, vomiting, visual disturbance, seizure (76% of patients exhibit three of these four signs), or even stroke [3-7].

The causes of HTN and hypertensive crisis vary by age. Primary HTN accounts for most hypertensive children over the age of six years, and 90% of the causes of HTN in children over 15 years of age [8-11]. Younger and more severe HTN pediatric patients are believed to account for secondary HTN. As a result of increasing mean BMI levels and increasing salt intake, the incidence of HTN in children appears to be steadily climbing [8,9,12]. The incidence of HTN in 2003 was reported to range from 1% to 5% of children aged 1 to 18 years in the United States [1,2,10]. In Taiwan, HTN has been found to range from 0.13% to 0.5% of children aged 6 to 15 years, and around 1% to 3% of school-aged children [13,14]. The objective of this study was to analyze the clinical features, etiology and treatment of children with first attacks of hypertensive crisis and to determine the predictors of severity of hypertensive crisis.

## Methods

### Patient population

From January 2000 to January 2008, we conducted this retrospective chart review of all patients 18 years and under with a diagnosis of HTN in our pediatric ED of Changhua Christian Hospital, a 2500-bed medical center in central Taiwan. The exclusion criteria were as follows: a BP below the 95^th^ percentile, a final diagnosis of transient hypertension, asymptomatic hypertensive patients, and those with incomplete data including inadequate body height or weight data, and no repeated BP measurements. A total of 112 patients presented to our pediatric ED with the diagnosis of primary and secondary hypertension. Sixteen patients were excluded for having a BP less than the 95^th^ percentile, 28 were excluded for asymptomatic hypertension, 10 were excluded due to a final diagnosis of transient hypertension, and three were excluded due to inadequate data. Therefore, the study group comprised 55 patients with hypertensive crisis. The study was approved by the Human Subjects Review Committee of the hospital.

The patients were divided into four age groups: infants (less than one year of age); preschool age (one to six years of age); elementary school age (seven to 12 years of age); and adolescents (13 to 18 years of age). Patients with hypertensive crisis were further subcategorized into two severity groups: hypertensive urgency and hypertensive emergency. Severity was based on the presence of end organ damage. Staging of HTN was defined as a BP between the 95^th^ percentile and 99^th^ percentile plus 5 mmHg (stage 1) and above the 99^th^ percentile plus 5 mmHg (stage 2).

### Blood pressure measurements

All children above three years of age received initial BP measurements at our pediatric ED when triaging. With the exception of children who were bedridden and infants who were unable to sit, BP was checked with the children in a seated position with their backs supported, feet on the floor, right arm supported, and with the cubital fossa at heart level. An appropriate cuff size was used with an inflatable bladder width that was at least 40% of the arm circumference at a point midway between the olecranon and the acromion. The cuff bladder length covered 80 to 100% of the circumference of the arm. Initially, aneroid manometers (automatic devices) were used to measure BP with an appropriate cuff. If the systolic BP (SBP) or diastolic BP (DBP) was higher than 120/80 mmHg, it was re-measured from both hands and legs [15]. BP measurements were performed every hour in the patients who presented with an unstable BP and in the patients requiring further observation. During the study period, the BP measurements were performed by different nurses, all of whom were well-trained and qualified.

### Identification of hypertension

HTN in children more than 12 months of age was defined according to BP standards based on gender, age and height as stipulated in the updated classification of hypertension by the National Blood Pressure Education Program Working Group on Hypertension in Children and Adolescents [7]. HTN was identified when the SBP or DBP was greater than or equal to the 95^th^ percentile; stage 1 HTN was defined as an SBP or DBP within the range of the 95^th^ percentile to the 99^th^ percentile plus 5 mmHg; stage 2 HTN was an SBP or DBP greater than the 99^th^ percentile plus 5 mmHg. For the patients younger than 12 months of age, hypertension was defined as an SBP or DBP greater than the 95^th^ percentile for infants of a similar age, size and sex according to a previously published report [16]. When systolic and diastolic percentiles differed, they were categorized according to the higher value. Transient HTN means transient blood pressure elevation caused by any emotional, painful, or uncomfortable events, and was defined as an asymptomatic BP higher than the 95^th^ percentile only once or twice, but returning to less than the 95^th^ percentile on the second or third measurement without any antihypertensive medication [5].

A hypertensive emergency was defined as HTN in the presence of acute or ongoing target-organ lesions, or HTN in relation to an immediate life-threatening event requiring immediate intervention to reduce the BP [9,11,13]. Hypertensive urgency was defined as an elevation in SBP/DBP higher than the 99^th^ percentile plus 5 mmHg with any complication related to the HTN and no evidence of target-organ lesions. End organ damage was defined as impairment in renal, myocardial, hepatic, and hematologic functions, and neurological manifestations derived from HTN. Acute (transient) end organ damage resulting from HTN was identified by abnormal clinical and laboratory findings which subsided after a decrease in BP. Abnormal data included abnormal electrocardiography findings, impaired renal function tests, elevated liver function markers, and neurological manifestations such as headache, altered consciousness and dizziness.

Hypertensive encephalopathy is a specific clinical syndrome characterized by acute neurological change in the setting of sudden and/or prolonged HTN that overcomes the autoregulatory capacity of the cerebral vasculature [17,18]. The syndrome is defined as severe hypertension in conjunction with symptoms of headache, altered mental status, seizure, or visual disturbances, and commonly presents with reversible posterior leukoencephalopathy seen on T2-weighted brain magnetic resonance images [19-22].

### Methods of analysis

The following data were collected and analyzed: age, gender, weight, height, family history of HTN, BP on arrival to the ED, clinical manifestations of hypertensive crisis (dizziness, headache, nausea/vomiting, visual symptoms, seizure/type, altered consciousness, chest tightness/pain, target-organ damage), reversibility, anti-hypertension drugs, underlying causes (renal disease, cardiovascular (CV), essential HTN, central nervous system (CNS) factors, endocrine/metabolic disorders, oncological disease), recurrent episodes, brain imaging and duration of hospitalization (ward/intensive care unit (ICU)). In addition, to decrease the influence of age, exact BMI percentile and z-score (standard deviation score), and SBP/DBP z-score according to the Center for Disease Control (CDC) growth charts were also analyzed.

CNS factors referred to CNS abnormalities as the cause of hypertension, which is different from hypertensive encephalopathy in causal connection. Essential hypertension was diagnosed after excluding secondary causes of hypertension by multiple tests, such as electrocardiography, metabolic panel, renal function tests, hemoglobin and urine routine tests, or other further specific tests including echocardiography, renal ultrasound, plasma rennin activity, plasma aldosterone, thyroid-stimulating hormone and 24-hour urine free cortisol.

Case distributions of hypertensive emergency and urgency were surveyed based on different time periods. During the study period, the BP levels, etiology, severity, and clinical manifestations were compared among children by age group and compared between the patients with hypertensive emergency and hypertensive urgency.

### Statistical analysis

All statistical analyses were performed using Fisher’s exact test, the Kruskal Wallis test, Jonckheere Terpstra test, and chi-square test as appropriate. The results of the descriptive analyses of independent variables were reported as percentages and mean ± S.D. A P value less than 0.05 was considered statistically significant. Statistical analyses were performed using SPSS software (version 15.0; SPSS Inc., Chicago, IL, USA).

## Results

### Characteristics of the study subjects

**Figure 1:**
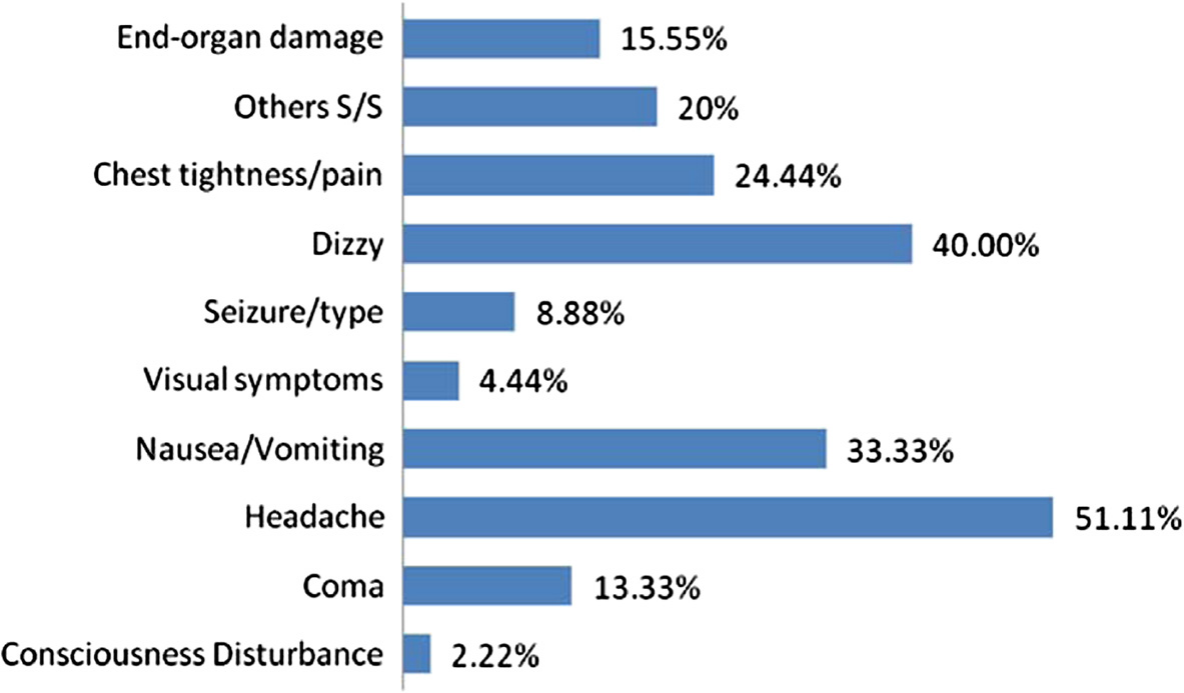
Ratios of clinical manifestations in the pediatric patients with hypertensive crisis.

From 2000 to 2007 55 children presented to the ED with hypertensive crisis, including 46 cases (83.6%) with hypertensive urgency and 9 cases (16.4%) with hypertensive emergency (incidence ratio 5:1). Five children had a diagnosis of hypertensive encephalopathy. The male-to-female incidence ratio was 5:1 (boys, n = 46; girls, n = 9). Most patients were in the adolescent group (n = 24, 43.6%). A family history of hypertension was only noted in the patients older than preschool age (n = 8, 14.5%). Almost all of the pediatric hypertensive crisis patients presented with hypertension stage 2 (n = 54, 98.1%). The major symptoms of hypertensive crisis were headache (n = 30, 54.5%), followed by dizziness (n = 25, 45.5%), and nausea/vomiting (n = 20, 36.4%) (Figure 1). The leading underlying causes were essential hypertension (n = 26, 47.2%), followed by renal disease, and endocrine/metabolic disease. The renal diseases included nephrotic syndrome (n = 2, 14.3%), IgA nephropathy (n = 2, 14.3%), poststreptococcal glomerulonephritis (n = 1, 7.1%), end stage renal disease (ESRD), Henoch-Schönlein purpura with glomerulonephritis (n = 1, 7.1%), ureteropelvic junction obstruction (n = 1, 7.1%), Alport syndrome with ESRD (n = 1, 7.1%), focal segmental glomerulosclerosis with ESRD (n = 1, 7.1%), polycystic kidney (n = 1, 7.1%), Alstrom syndrome with chronic renal insufficiency (n = 1, 7.1%), inborn error, hyperammonemia with ESRD (n = 1, 7.1%), ESRD s/p renal transplantation (n = 1, 7.1%), and SLE with lupus glomerulonephritis (n = 1, 7.1%). The endocrine and metabolic diseases included hyperthyroidism (n = 3, 33.3%), diabetes mellitus (n = 3, 33.3%), hyperaldosteronism (n = 1, 11.1%), adrenal hyperplasia (n = 1, 11.1%), and methylmalonic academia with hyperuricemia (n = 1, 11.1%). The oncological disorders included pheochromocytoma associated with neurofibromatosis (n = 1, 50%) and paraganglioneuroma (n = 1, 50%). The recurrence rate of hypertensive crisis was 29.1% (16 cases: 12 urgency; 4 emergency) during the study period. A total of 33 (60%) patients who visited the ED were hospitalized: 24 to wards, 7 to the pediatric intensive care unit (PICU), and 2 to the pediatric observation unit (POU) of the pediatric ED.

### Hypertensive crisis by age group

Boys had a higher morbidity of hypertensive crisis in every age group except for the infant group (Table 1). A positive family history was present only in the children older than 7 years: 2 (10.5%) in the school age group, and 6 (25%) in the adolescent group. About half of the patients had underlying causes (n = 27, 49%). Essential HTN was also diagnosed in approximately half of the patients (n = 26, 47.3%). Among the underlying causes, essential HTN had a significant correlation with age (Table 1). The z-scores of BMI and SBP/DBP in the children by age group are listed in Table 2. The mean BMI values of the children with hypertensive crisis were all above the threshold of obesity. The mean SBP/DBP in the patients with hypertensive crisis was 161/102 mmHg. In contrast to DBP, SBP had a significant trend with older age. The patients with hypertensive crisis received antihypertensive agents, and the BP levels gradually decreased by about 25% to 30% within one hour, finally returning to normal ranges about two to three days later during hospitalization.

**Table 1:** Characteristics of the patients with hypertensive crisis by age group

	**Age (years)**									
	**<1 (n = 7)**		**1–6 (n = 5)**		**7–12 (n = 19)**		**13–18 (n = 24)**			
**Variables**	**N**	**%**	**N**	**%**	**N**	**%**	**N**	**%**	**P-value**^**1**^	**P-value**^**2**^
**Gender**										
Female	0	0.0	3	60.0	4	21.1	2	8.3	0.037	0.517
Male	7	100.0	2	40.0	15	78.9	22	91.7		
Family history	0	0.0	0	0.0	2	10.5	6	25.0	0.362	0.049
**Blood Pressure**										
SBP > 99th percentile +5	7	100.0	4	80.0	19	100.0	24	100.0	0.091	0.282
DBP > 99th percentile +5	6	85.7	3	60.0	14	73.7	19	79.2	0.779	0.955
Stage of hypertension										
Stage 1	0	0.0	1	20.0	0	0.0	0	0.0	0.108	0.313
Stage 2	7	100.0	4	80.0	19	100.0	24	100.0		
**Clinical presentations**										
Altered Consciousness	2	28.6	2	40.0	2	10.5	3	12.5	0.068	0.372
Headache	0	0.0	3	60.0	13	68.4	14	58.3	0.085	0.054
Nausea/Vomiting	2	28.6	2	40.0	8	42.1	8	33.3	0.922	0.960
Visual symptoms	0	0.0	0	0.0	0	0.0	2	8.3	0.693	0.201
Seizure	1	14.3	2	40.0	1	5.3	2	8.3	0.142	0.282
Dizzy	2	28.6	0	0.0	11	57.9	12	50.0	0.094	0.130
Chest pain	0	0.0	1	20.0	8	42.1	7	29.2	0.195	0.187
End-organ damage	2	28.6	2	40.0	3	15.8	2	8.3	0.178	0.086
**Reversibility**	3	42.9	3	60.0	9	47.4	13	54.2	0.931	0.701
**Anti-HTN drugs**	2	28.6	2	40.0	10	52.6	16	66.7	0.285	0.054
**Etiology**										
Essential HTN	0	0.0	1	20.0	8	42.1	15	62.5	0.008	0.001
Renal disease	3	42.9	1	20.0	6	31.6	4	16.7	0.423	0.166
CNS	2	28.6	1	20.0	0	0.0	1	4.1	0.202	0.120
Endocrine/metabolic	1	14.3	1	20.0	3	15.8	4	16.7	0.952	0.358
CV	1	14.3	0	0.0	1	5.3	0	0.0	0.252	0.117
Oncology	0	0.0	1	25.0	1	5.3	0	0.0	0.092	0.389
**Recurrent episode**	4	57.1	1	20.0	5	26.3	6	25.0	0.440	0.196
**Severity**										
Urgency	5	71.4	3	60.0	16	84.2	22	91.7	0.178	0.086
Emergency	2	28.6	2	40.0	3	15.8	2	8.3		
**Hospitalization**										
Ward	3	42.9	4	80.0	8	42.1	9	37.5	0.425	0.398
ICU	0	0.0	1	20.0	3	15.8	3	12.5	0.271	0.886
POU	4	57.1	0	0.0	8	42.1	12	50.0	1.000	0.565

^1^by Fisher’s exact test.

^2^by the chi-square test for trend.

SBP: systolic blood pressure; DBP: diastolic blood pressure; HTN: hypertension; CNS: central nervous systems; CV: cardio-vascular; ICU: intensive care unit; POU: pediatric observation unit.

**Table 2:** **Description of results obtained in various age categories of hypertensive crisis patients in characteristics**, **BMI and blood pressure**

**Age**	**1–6 (n = 3)**^**a**^	**7–12 (n = 13)**^**b**^	**13–18 (n = 18)**^**c**^	**P-value**	**Post hoc tests**
	**Mean ± SD**	**Mean ± SD**	**Mean ± SD**		
BW	27.67 ± 20.43	53.35 ± 28.19	83.64 ± 33.27	0.005	a,b < c
Height	103.00 ± 15.72	144.66 ± 10.38	167.95 ± 9.58	<0.001	a < b < c
Height Z-Score	−8.85 ± 2.91	−3.43 ± 1.15	−0.70 ± 1.24	<0.001	a < b < c
BMI	24.30 ± 12.95	24.88 ± 12.60	29.05 ± 9.08	0.522	
BMI Z-score	1.52 ± 1.68	1.13 ± 1.49	3.07 ± 6.96	0.587	
BMI Percentile	81.33 ± 20.74	75.54 ± 31.11	84.56 ± 25.93	0.675	
SBP	189.67 ± 36.91	158.38 ± 25.09	164.22 ± 23.84	0.174	
DBP	136.00 ± 38.16	93.54 ± 19.98	104.61 ± 19.03	0.013	a > b
Expected SBP	103.80 ± 16.23	95.17 ± 4.12	109.91 ± 5.11	<0.001	b < c
Expected DBP	76.78 ± 22.76	54.46 ± 3.42	62.32 ± 3.76	<0.001	a > c > b
SBP Z-score	8.10 ± 4.15	5.93 ± 2.34	5.08 ± 2.17	0.135	
DBP Z-score	5.22 ± 5.26	3.41 ± 1.74	3.68 ± 1.75	0.432	

a,b,c: the mean data of each age group.

P-value by one-way analysis of variance followed by Sidak multiple comparisons at a type I error of 0.05.

SBP: systolic blood pressure; DBP: diastolic blood pressure; BMI: body mass index.

### Patients with hypertensive encephalopathy

Five male patients, all without a family history of hypertension, had hypertensive encephalopathy at the ages of 5, 9, 12, 13 and 14 years, respectively (Table 3). Their presenting BP levels at the ED were all classified as stage 2 hypertension, and four of them had a DBP and SBP above the 99^th^ percentile plus 5 mmHg, ranging from 148 to 231 mmHg of systolic BP, and 86 to 172 mmHg of diastolic BP. All had altered consciousness; three were in a coma on arrival and recovered after their BP had been controlled. The major associated symptoms were headache and nausea/vomiting. Oncological causes were the major factors in the patients with hypertensive encephalopathy, one being induced by pheochromocytoma and one by paraganglioneuroma. Two of the patients with hypertensive encephalopathy had recurrent hypertensive crisis episodes during the study period. Magnetic resonance imaging (MRI) of the brain revealed increased signal intensity in the subcortical white matter and cortical gray matter of the parieto-occipital area, cerebellum and basal ganglia. Magnetic resonance spectrometry (MRS) showed a high lactate peak with normal N-acetyl aspartate (NAA), choline and creatine levels (Table 3).

**Table 3:** The characteristics of the patients with hypertensive encephalopathy (N = 5)

	**Case 1**	**Case 2**	**Case 3**	**Case 4**	**Case 5**
Gender (F/M)	M	M	M	M	M
Age (year)	9	12	13	5	14
Weight (kg)	24	39	68	19	35
Height (cm)	134		158	110	
Family history	no	no	no	no	no
Arrival BP	166/130	176/86	220/128	231/172	148/109
Hypertension stage	stage 2	stage 2	stage 2	stage 2	stage 2
SBP > 99^th^ percentile + 5	33(25.3%)	42(31.4%)	82(59.4%)	106(84.8%)	3(2%)
DBP > 99^th^ percentile + 5	38(41.3%)		34(36.1%)	86(101%)	12(11%)
clinical manifestations					
Consciousness change	Coma	Coma	Coma	Drowsy	Disturbance
Headache	+	0	+	+	+
Nausea/Vomiting	0	0	+	+	+
Visual symptoms	0	0	+	0	0
Seizure	0	0	0	+	+
Dizzy	0	0	0	0	+
Chest tightness	0	0	0	0	0
Drug for anti-HTN	Labetalol, Furosemide	Labetalol	Nifedipine, Labetalol	Nifedipine	captopril / amlodipine
Underlying causes	oncology Pheochromocytoma	0	0	oncology Paraganglioneuroma	Renal disease
Recurrent episode (times)	0	0	0	3	>5
CSF data	0	0	0	0	normal
EEG finding	0	0	normal	0	normal
Hospitalization duration (days) (ward/ICU/POU)	9(4/5/0)	11(5/6/0)	5(3/2/0)	7(7/0/0)	6(6/0/0)

SBP: systolic blood pressure; DBP: diastolic blood pressure; HTN: hypertension; CSF: cerebrospinal fluid; EEG: Electroencephalography; ICU: intensive care unit; POU: pediatric observation unit.

### Case distribution analysis and treatment

**Figure 2:**
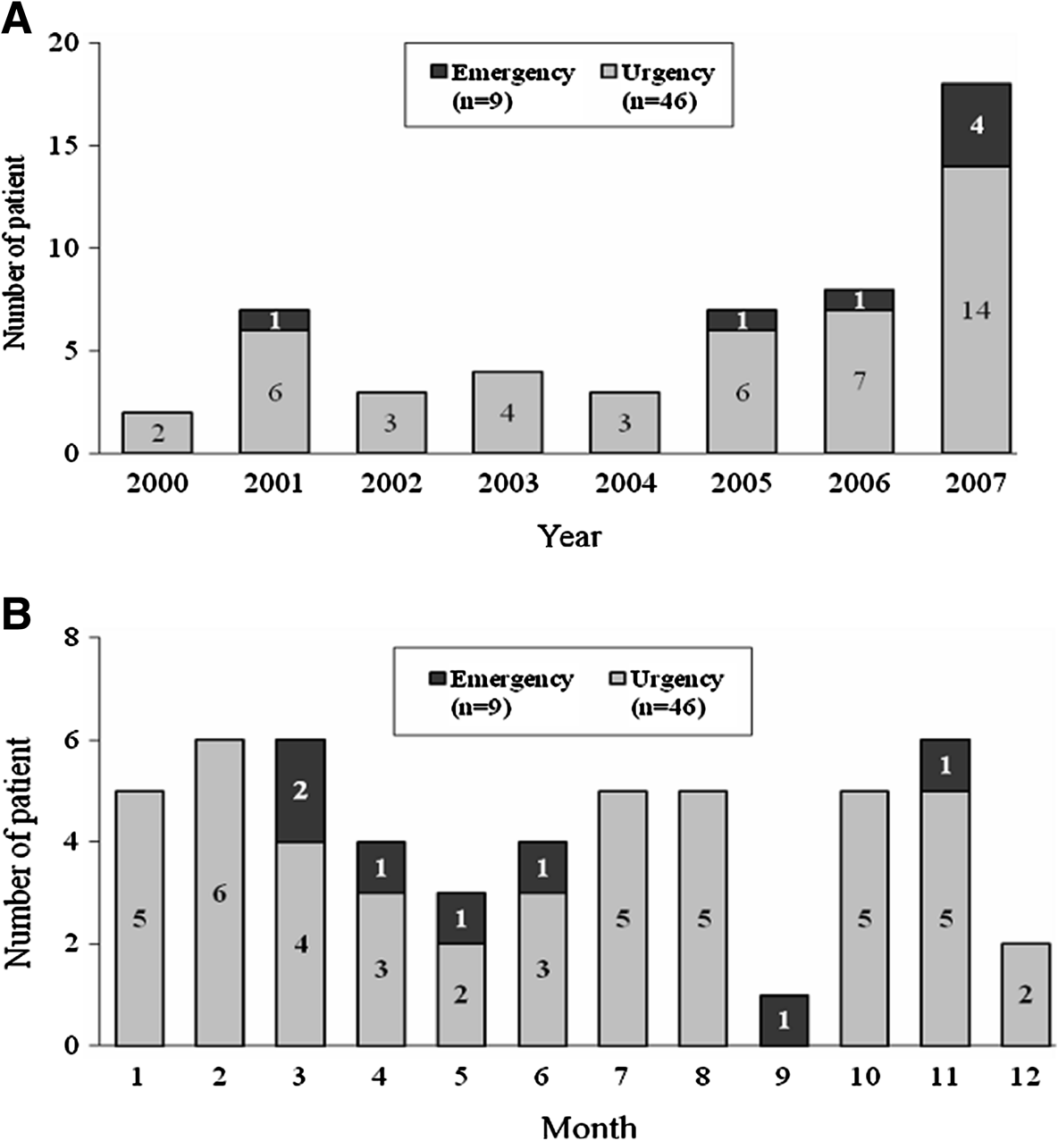
**Distribution of hypertensive crisis in the children from 2000 to 2008 by year** (**A**), **and month** (**B**).

The year and month distribution analysis of hypertensive urgency and emergency is shown in Figure 2. The analysis revealed that the prevalence of children with hypertensive crisis in the total number of children who came to the ED increased by year during the study period. The distribution by month revealed that hypertensive emergency occurred mostly in the spring (March to June).

During the study period, there were no cases of mortality or sequelae. Nine patients received multi-antihypertensive agents. Long-term-acting amlodipine besylate was used in seven patients; atenolol in nine patients; captopril in six patients; nifedipine in four patients; carvedilol, inderol and labetalol were used separately in three cases; and pentoxifylline, servidipine, lisinopril, and nicametate were each used once. None of the patients received antihypertensive medication before arriving at the pediatric ED.

## Discussion

In comparison to adults, hypertensive crisis in children is a relatively rare condition. It presents with elevated BP and related symptoms and is potentially life-threatening. In clinical practice, high BP is often treated as an associated symptom rather than a specific complaint. However, the importance of pediatric hypertension is easily underestimated without understanding the damage caused by high BP.

For the pediatric patients with hypertensive crisis in our study, as their age increased, more related family histories of HTN and more essential hypertensive causes were noted. After seven years of age, essential HTN became the major cause of first-attack hypertensive crisis, whereas before the age of seven, hypertensive crisis was mostly attributed to secondary HTN. However, even though secondary hypertension was the major cause for the younger patients, there was no statistical correlation between age and different underlying causes of pediatric hypertensive crisis. Renal diseases were the major underlying cause of first-attack hypertensive crisis, and they could induce a first attack of hypertensive crisis at any age. Catecholamine producing tumors, such as pheochromocytoma and paraganglioneuroma induced higher blood pressure, resulting in the highest BP and most severe clinical outcomes, and were able to induce first-attacks of hypertensive emergency and even hypertensive encephalopathy at any age.

Almost all of the patients in this study presented with BP levels higher than the 99^th^ percentile plus 5 mmHg (stage 2 HTN). Therefore, the 99^th^ percentile plus 5 mmHg may serve as a critical threshold for a high risk of hypertensive crisis in children. Some studies have also suggested that stage 2 hypertension requires prompt evaluation and treatment once the stage of HTN is persistent [1,6]. Moreover, symptoms such as headache and nausea/vomiting associated with a BP level above the 99^th^ percentile plus 5 mmHg should be regarded as warning signs of the end organ damage and secondary hypertension. Other reported related symptoms of HTN in children include blurred vision, and disease-specific symptoms such as edema, pallor and petechiae [23]. In addition, although the patients with hypertensive emergency in the current study did not have any sequelae, permanent neurological damage, blindness, and chronic renal failure have been reported to be long-term consequences of hypertensive emergency [24].

There are some limitations to our study. First, due to the relatively low incidence of hypertensive crisis, only 55 patients were identified over the eight-year period of this study. The small sample size and the selection of participants from a single medical center limit the generalizability of our results to the entire population of patients with hypertensive crisis. Second, family histories and physical examination findings are not easily identifiable in a retrospective study, and this may have led to missing data in the analysis. These limitations may have led to bias in analyzing the first attacks of hypertensive crisis in the ED.

## Conclusions

In conclusion, ED physicians should pay attention to all pediatric patients who present with an elevated BP and clinical symptoms including headache, nausea/vomiting, and altered consciousness. Once patients have a BP level higher than stage 2 hypertension, appropriate and immediate antihypertensive medications are necessary to prevent further damage.

## Competing interests

The authors declare that they have no competing interests.

## Authors’ contributions

WCY and CYC reviewed the medical records, analyzed and interpreted the data, and drafted the manuscript; LLZ and YW interpreted the data, and drafted the manuscript. YJC analyzed and interpreted the data. HPW designed and oversaw the study, interpreted the data, and revised the manuscript. All authors have read and approved the manuscript for publication.

## Pre-publication history

The pre-publication history for this paper can be accessed here:

http://www.biomedcentral.com/1471-2431/12/200/prepub
